# Hydrogen Sulfide Inhibits Enzymatic Browning of Fresh-Cut Chinese Water Chestnuts

**DOI:** 10.3389/fnut.2021.652984

**Published:** 2021-06-04

**Authors:** Yuan Dou, Chunmei Chang, Jing Wang, Zhipeng Cai, Wei Zhang, Huaying Du, Zengyu Gan, Chunpeng Wan, Jinyin Chen, Liqin Zhu

**Affiliations:** ^1^College of Food Science and Engineering, Jiangxi Agricultural University, Nanchang, China; ^2^Jiangxi Key Laboratory for Postharvest Technology and Nondestructive Testing of Fruits and Vegetables, Collaborative Innovation Center of Postharvest Key Technology and Quality Safety of Fruits and Vegetables, Jiangxi Agricultural University, Nanchang, China; ^3^College of Agronomy, Jiangxi Agricultural University, Nanchang, China; ^4^College of Materials and Chemical Engineering, Pingxiang University, Pingxiang, China

**Keywords:** hydrogen sulfide, fresh-cut Chinese water chestnuts, browning, antioxidant defense system, phenolic metabolic activity

## Abstract

This work investigates the role of hydrogen sulfide (H_2_S) in the browning and regulating the antioxidant defensive system in fresh-cut Chinese water chestnuts. The samples were fumigated with 0, 10, and 15 μl L^−1^ of H_2_S and stored at 10°C for 8 days. The results indicated that the H_2_S treatment significantly inhibited the browning of fresh-cut Chinese water chestnuts, reduced superoxide anion (${\text{O}}_{2}^{\cdot -}$) production rate and H_2_O_2_ content accumulation, promoted the increase of total phenol content, and enhanced activities of catalase (CAT), superoxide dismutase (SOD), ascorbate peroxidase (APX), and glutathione reductase (GR) (*P* < 0.05). On the other hand, phenylalanine ammonia lyase (PAL), polyphenol oxidase (PPO), and peroxidase (POD) activities remained at a low level in the H_2_S treatment (*P* < 0.05). This result suggested that H_2_S treatment might be a promising approach to inhibit browning and prolong the shelf life by enhancing oxidation resistance and inhibiting browning enzyme activity of fresh-cut Chinese water chestnuts during storage. Among them, the 15 μl L^−1^ H_2_S treatment had the best effect on fresh-cut Chinese water chestnuts.

## Introduction

The Chinese water chestnuts (CWCs; *Eleocharis tuberosa*) are widely grown in China and are rich in starch, minerals, vitamins, and protein. They are a popular aquatic plant with special taste and high medicinal values (1, 2). The CWCs are small in size and wrapped in hard shells; they are quite difficult to peel off; attached to their skin are plenty of bacteria and/or microbial eggs, which bring inconvenience to the consumers. With the development of the ready-to-eat food industry, fresh-cut CWCs can greatly meet the needs of consumers. However, after being peeled, they will not only suffer serious mechanical damage but also be prone to discoloration, which will affect the edible quality and reduces their shelf life and commercial value (3). At present, there are two viewpoints on the discoloration of fresh-cut CWCs: browning and yellowing. In the experiment of treating fresh-cut CWCs with eugenol emulsion, the browning inhibition mechanism related to enzyme activity and polyphenol substrate was studied (4). Another study also found that the yellowing substances on the surface of fresh-cut CWCs were mainly flavonoids such as naringenin and eriodictyol (2). Therefore, certain studies reported different treatments for fresh-cut CWCs to prolong their shelf life. Peng and colleagues treated fresh-cut CWCs with different concentrations of H_2_O_2_ and found that H_2_O_2_ treatment could inhibit browning enzyme activities and could maintain the nutritional value of samples (5). N_2_ treatment induced antioxidant enzyme activity and antioxidant content and delayed the spoilage of fresh-cut CWCs (6). Ferulic acid was also reported to suppress the activity of browning enzymes during storage period and to slow down the changes in color (7).

Recently, after nitric oxide (NO) and carbon monoxide (CO), H_2_S is the latest endogenous signaling molecule, and it is observed that low concentrations of H_2_S can play active roles in biological systems. Moreover, many studies have shown that H_2_S can inhibit postharvest senescence of fruits and vegetables as well as improve their commercial value (8). Hu and co-workers investigated and proved that H_2_S treatment maintained the nutrients levels in the strawberry fruit, significantly inhibiting the respiratory rate and reducing the accumulation of reactive oxygen species (ROS) with the improved antioxidant capacity of the strawberry fruit (9). Our previous study elucidated the effect of H_2_S treatment on shelf life of kiwifruit after harvest where H_2_S treatment could eliminate the accumulation of ROS by increasing the activity of antioxidant enzymes, thus delaying the maturation of kiwifruits (10). In another study, H_2_S treatment in broccoli can maintain a high level of metabolites, can inhibit the increase of browning enzymes, and can play a role in regulating aging of broccoli (11). H_2_S has been applied to inhibit postharvest senescence in mulberry fruit (12), water spinach (13), banana fruit (14), grape (15), hawthorn fruit (16), pak choy (17), peach fruit (18), and kiwifruits (19).

In searching for effective anti-browning treatment for fresh-cut CWCs, we fumigated with H_2_S gas for 30 min, and H_2_S was proved to prevent discoloration of fresh-cut CWCs. The current study aimed to investigate the effect of H_2_S on browning fresh-cut CWCs and the regulation of oxidation resistance and phenolic metabolic activity.

## Materials and Methods

### Sample Preparation

CWCs [*Eleocharis dulcis* (Burm. f.) Trin.] were obtained from a commercial market in Nanchang City, Jiangxi Province of China. Before treatment, the evenly sized CWCs were selected, pre-cooled at 2°C for 24–48 h, and then washed and peeled using a knife. Samples were put into a sealed glass container, with injected H_2_S gas (purity of 99.99% and concentration of 0, 10, and 15 μl L^−1^) into the glass container (30 L) through a rubber stopper with a syringe, and then fumigated for 30 min. The control group (CK) was treated with air. After fumigation, all samples were placed in polypropylene plastic boxes (size: 150 × 210 × 25 mm), wrapped with PE cling film, and stored at 10°C. Each treatment contained about 1,500-g fresh-cut CWCs and was replicated three times. Six samples every 2 days were got out for color analysis, and six other per replicate were mixed and frozen with liquid nitrogen stored in a refrigerator at −80°C for further measurement.

### Measurement of Browning Index

The color change of fresh-cut CWCs was analyzed using a Chromatic meter equipped (ColorQuest XE) with a measuring head. The browning of the CWCs surface was measured by the changes in the L^*^, a^*^, and b^*^ parameters. The browning index (BI) is calculated according to the following formulas (20):

(1) $$\begin{array}{r} BI=\frac{100*\left(x-0.31\right)}{0.172} \end{array}$$

(2) $$\begin{array}{r} x=\frac{a^{*}+1.75L^{*}}{5.645L^{*}+a^{*}-3.012b^{*}} \end{array}$$

### Measurement of Total Phenolic Content

The total phenolic content was measured as stated by the Folin–Ciocalteu procedure, with some modifications (21). Samples of 1.0 g were homogenized with ethanol. After centrifugation, 1 ml of supernatants, 1 ml of Folin–Ciocalteu reagent, 5 ml of 5% NaCO_3_, and 18 ml of distilled H_2_O were mixed and incubated for 60 min. The absorbance at 760 nm was determined using a spectrophotometer, and the results were expressed as mg g^−1^ fresh weight (FW).

### Determination of Reactive Oxygen Species

${\text{O}}_{2}^{\cdot -}$ production rate was determined with the method of Zhu et al. (10), with some modifications. Samples (0.1 g) was homogenized in 1 ml of 50 mmol L^−1^ phosphate buffer (pH 7.8) and centrifuged at 12,000 *g* for 10 min. The supernatant (0.5 ml) was mixed with 1 ml of 65 mmol L^−1^ phosphate buffer (pH 7.8) and 0.5 ml of 10 mmol L^−1^ hydroxylammonium chloride and then incubated for 20 min at 25°C. The incubation solution (0.5 ml) was then mixed with 0.5 ml of 7 mmol *a*-naphthylamine and 1 ml of 17 mmol 4-aminobenzene sulfonic acid for a further 30 min. Five milliliters of *n*-butanol was added into the reaction mixture, and then the *n*-butanol phase was used for the determination of ${\text{O}}_{2}^{\cdot -}$. ${\text{O}}_{2}^{\cdot -}$ production rate was expressed as nmo1 min^−1^ g^−1^ FW.

For H_2_O_2_ determination, 0.1 g of fresh-cut CWCs was homogenized with 1 ml of cold 100% acetone and centrifuged at 12,000 *g* for 15 min (4°C). The supernatant was collected for H_2_O_2_ analysis by the method of Patterson et al. (22). The absorbance at 415 nm was measured using a spectrophotometer and expressed as μmo1 g^−1^ FW.

### Enzyme Activities

For catalase (CAT) and glutathione reductase (GR), fresh-cut CWCs (0.1 g) were homogenized in 1 ml of 0.2 mol L^−1^ phosphate buffer (pH 6.5) containing 0.5 g of polyvinylpyrrolidone (PVP). CAT activity was determined according to Ren et al. (23), with some modifications. The homogenate was centrifuged at 12,000 *g* for 10 min at 4°C, and the supernatant was used as crude enzyme solution. As substrates, 2.8 ml of 40 mmol L^−1^ H_2_O_2_ (dissolved with 50 mmol L^−1^ of sodium phosphate buffer, pH 7.0) was added into 0.2 ml of enzyme solutions. The disappearance of H_2_O_2_ was monitored by recording the decrease in absorbance at 240 nm. For GR, 1 ml of reaction solutions included 50 mM of potassium phosphate buffer (pH 7.8), 0.2 mM of NADPH, 2.5 mM of GSSG (glutathione disulfide), and 50 μl of cellular enzyme extract. GR activity was determined based on the oxidation of NADPH at 340 nm (8).

Ascorbate peroxidase (APX) activity was determined according to Chen et al. (8), with some modifications. For APX activity, 0.1 g of fresh-cut CWCs was homogenized in 1 ml of 0.05 mol L^−1^ phosphate buffer (pH 7.8) containing 2 mmol L^−1^ of ascorbic acid and 0.5 mmol L^−1^ of EDTA. Five hundred microliters of supernatant was mixed with 2 ml of 0.5 mmol L^−1^ phosphate buffer (pH 7.5), 1 ml of 1 mmol L^−1^ ascorbic acid, and 1 ml of 30% H_2_O_2_ (v/v), and then absorbance was determined at 290 nm.

Superoxide dismutase (SOD) activity was determined according to Yin et al. (24), with some modifications. For SOD activity, samples (0.1 g) were homogenized in 1 ml of 50 mmol L^−1^ phosphate buffer (pH 7.8) and centrifuged; and 0.5 ml of collected supernatants was mixed with 0.5 ml of 20 μmol L^−1^ riboflavin, 750 μl of NBT, 0.5 ml of 100 mmol L^−1^ MET, and 0.5 ml of 100 μmol L^−1^ EDTA; the SOD enzyme activity was determined at 560 nm using a spectrophotometer.

The determination of polyphenol oxidase (PPO) and peroxidase (POD) activities refers to the method of Min et al. (25), with some modifications. Fresh-cut CWCs (0.1 g) were homogenized in 1 ml of different pre-cooled sodium phosphate buffer and then centrifuged at 12,000 *g* for 15 min at 4°C. For PPO determination, the assay consisted of treated enzyme extract, pre-cooled sodium phosphate buffer, and catechol solution to determine PPO activity at 420 nm using a spectrophotometer. For POD determination, the clear supernatants were collected and detected. The assay mixture consisted of enzyme solution, sodium phosphate buffer (pH 6.5), H_2_O_2_, and guaiacol; the PPO enzyme activity was determined at 470 nm using a spectrophotometer.

Phenylalanine ammonia lyase (PAL) activity was determined with the method of Lu et al. (26), with some modifications. Samples of fresh-cut CWCs (0.1 g) were ground in 0.1 mol L^−1^ of sodium borate buffer (pH 8.8) containing 0.02 mol L^−1^ of β-mercaptoethanol, 2 mmol L^−1^ of EDTA, and 1.0 g of PVP and centrifuged at 4°C for 15 min at 12,000 *g*. Supernatant of 0.1 ml was mixed with 1 ml of phenylalanine and incubated at 37°C for 60 min; and then the absorbance was detected at 290 nm.

All enzyme activities were expressed as U g^−1^ FW.

### Statistical Analysis

SPSS software package Version 18.0 was used for statistical analysis. Statistical significance was tested by one-way analysis of variance (ANOVA), and values were expressed as means ± standard deviation (SD) (*n* = 3). Duncan's test (*P* < 0.05) was used to determine the significance in the differences.

## Results

### Change of Browning Index

**Figure 1:**
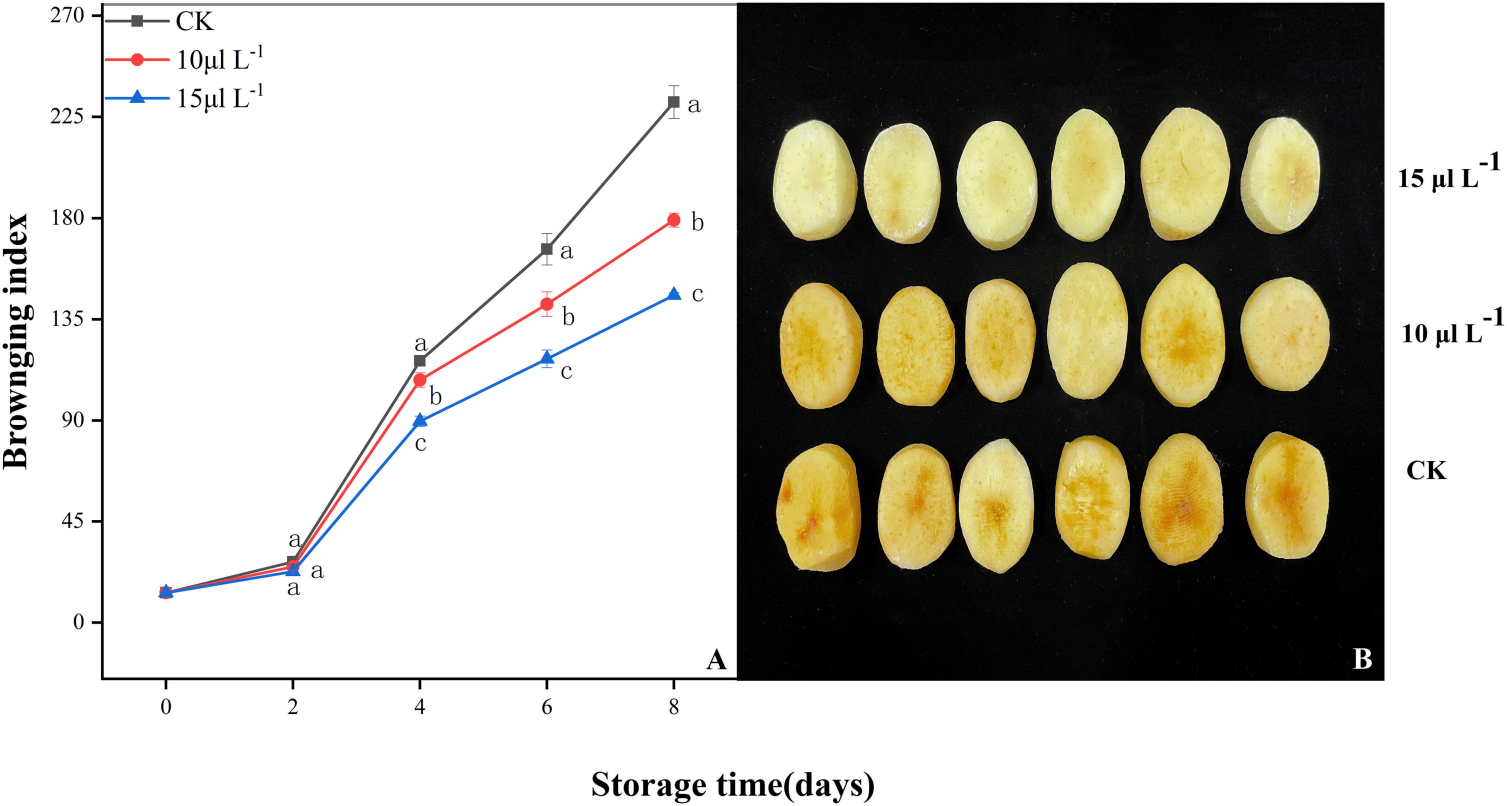
Effects of H_2_S treatment on browning index **(A)** and photograph (4 days) **(B)** of fresh-cut Chinese water chestnuts during storage at 10°C for 8 days. Vertical bars represent the standard errors of the means. Different letters (at the same day) mean that they were significantly different (Duncan's multiple range test, *P* < 0.05).

In all treatments, a continual increase in BI of fresh-cut CWCs was observed throughout storage period (Figure 1A). Low BI was found with the H_2_S treatment group (*P* < 0.05); it is worth noting that the 15 μl L^−1^ H_2_S treatment maintained a lower level during storage (*P* < 0.05). At the fourth day, the effect of H_2_S on inhibiting browning of samples was observed and presented in Figure 1B.

### Changes in Reactive Oxygen Species

**Figure 2:**
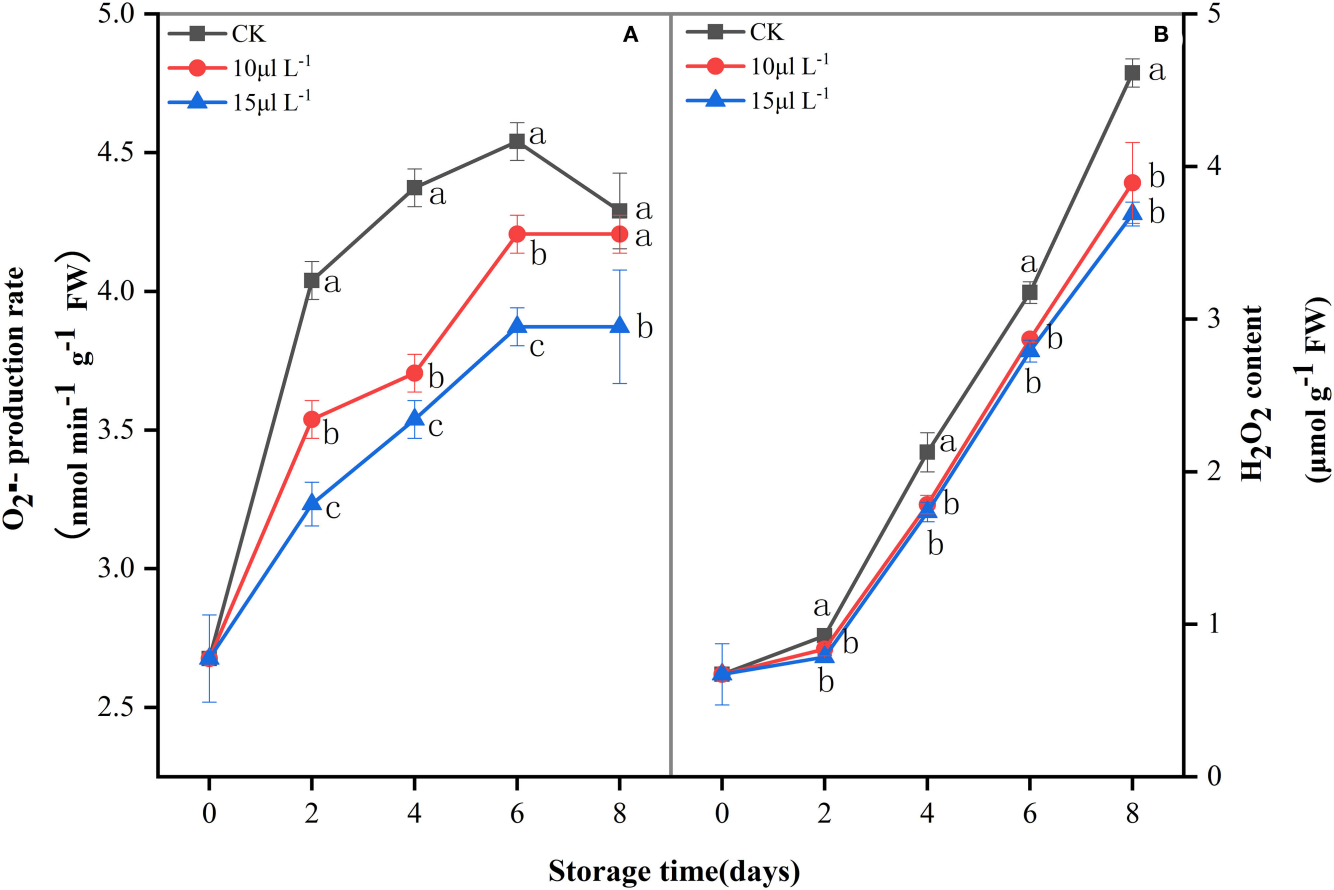
Effects of H_2_S treatment on ${\text{O}}_{2}^{\cdot -}$ production rate **(A)** and H_2_O_2_ content **(B)** of fresh-cut Chinese water chestnuts during storage at 10°C for 8 days. Vertical bars represent the standard errors of the means. Different letters (at the same day) mean that they were significantly different (Duncan's multiple range test, *P* < 0.05).

ROS (${\text{O}}_{2}^{\cdot -}$) production rate showed an upward trend of fresh-cut CWCs during the whole storage period (Figure 2A), whereas the 15 μl L^−1^ H_2_S treatment significantly inhibited ${\text{O}}_{2}^{\cdot -}$ production rate (*P* < 0.05). H_2_O_2_ content increased slowly in the first 2 days and increased rapidly afterwards (Figure 2B). Compared with the control, H_2_S treatment significantly reduced the content of H_2_O_2_ during storage (*P* < 0.05).

### Effect of H_2_S on Antioxidant Enzyme Activities

**Figure 3:**
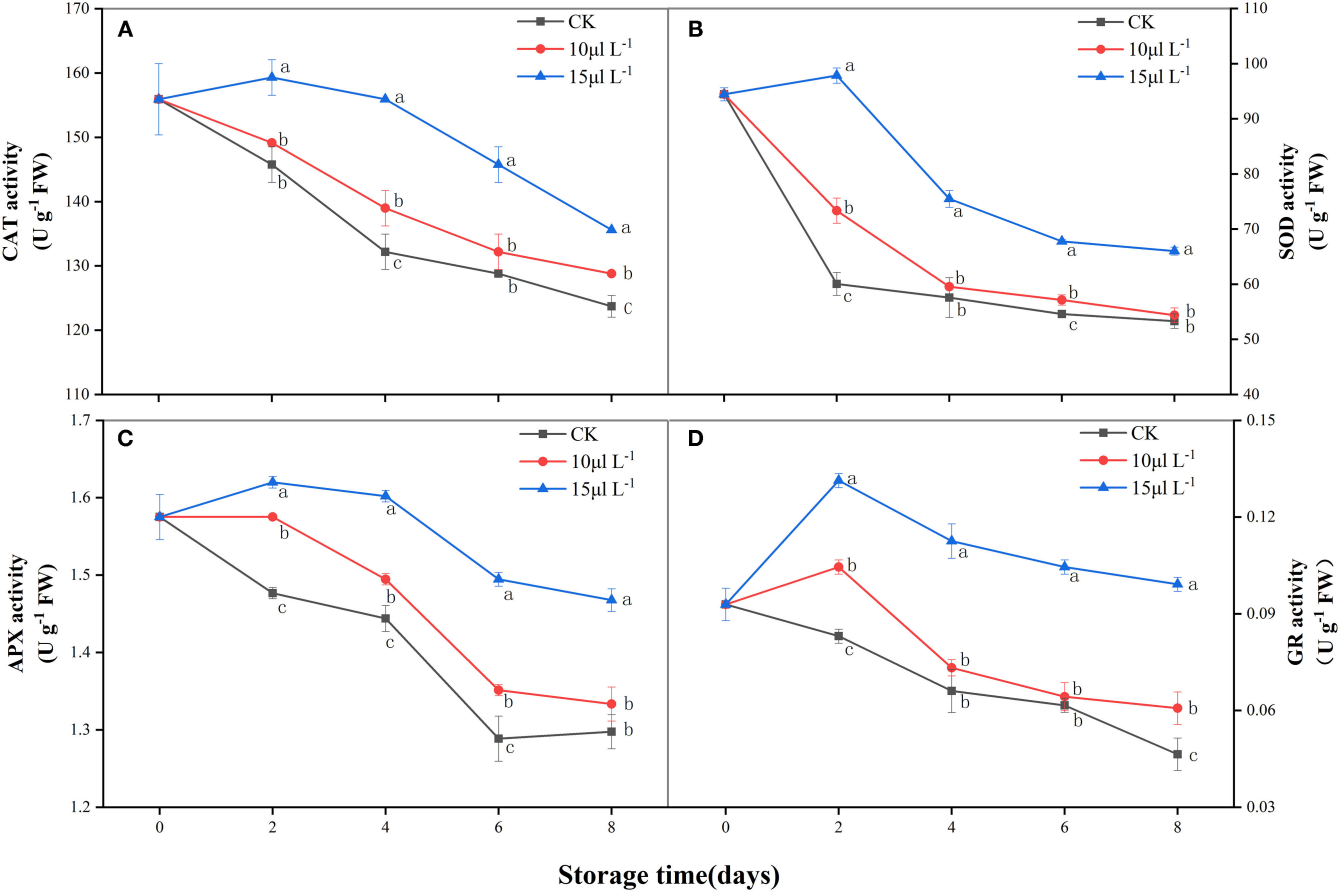
Effects of H_2_S treatment on CAT **(A)**, SOD **(B)**, APX **(C)**, and GR **(D)** activities of fresh-cut Chinese water chestnuts during storage at 10°C for 8 days. Vertical bars represent the standard errors of the means. Different letters (at the same day) mean that they were significantly different (Duncan's multiple range test, *P* < 0.05). CAT, catalase; SOD, superoxide dismutase; APX, ascorbate peroxidase; GR, glutathione reductase.

CAT activity of the control group and the 10 μl L^−1^ H_2_S treatment group decreased rapidly over 8 days. The 15 μl L^−1^ H_2_S treatment increased in the first 2 days and then decreased, and its activity was maintained at a significantly higher level than that of other groups (*P* < 0.05) (Figure 3A). Similar with CAT, the SOD activity of the control group and the 10 μl L^−1^ H_2_S treatment group in samples also decreased rapidly during storage. At day 8, SOD activity of the 15 μl L^−1^ H_2_S treatment group was 1.24-fold higher than that of the control group (*P* < 0.05) (Figure 3B). The activity of APX in all treatments showed on a fluctuant decreasing process (Figure 3C). Compared with the control, 1.13-fold higher level of APX activity was detected in the 15 μl L^−1^ H_2_S treatment on day 8 (*P* < 0.05). GR activity of the control group decreased rapidly during storage (Figure 3D). H_2_S fumigation of 15 μl L^−1^ induced a burst of GR activity on 2 days and maintained significantly higher GR activity than the control group in the whole storage period (*P* < 0.05).

### Effect of H_2_S on Total Phenolic Content and Browning Enzyme Activities

**Figure 4:**
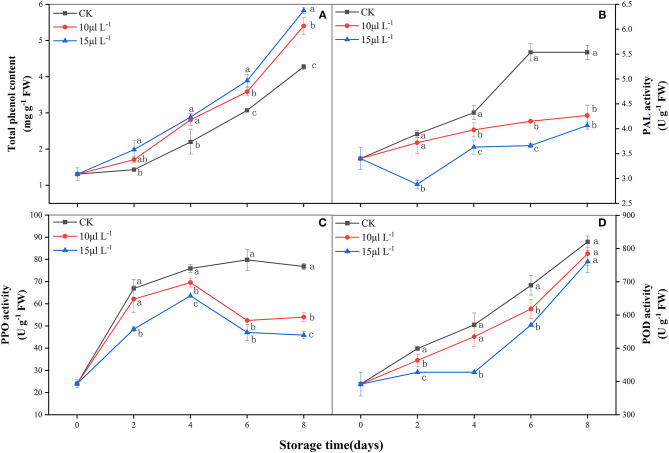
Effects of H_2_S treatment on total phenolic content **(A)**, PAL **(B)**, PPO **(C)**, and POD **(D)** activities of fresh-cut Chinese water chestnuts during storage at 10°C for 8 days. Vertical bars represent the standard errors of the means. Different letters (at the same day) mean that they were significantly different (Duncan's multiple range test, *P* < 0.05). PAL, phenylalanine ammonia lyase; PPO, polyphenol oxidase; POD, peroxidase.

During storage, there was an increase in total phenolic content of fresh-cut CWCs in all treatments (Figure 4A); the 15 μl L^−1^ H_2_S treatment demonstrated a higher level than did other groups during the later stage of storage (*P* < 0.05). PAL activity of the 15 μl L^−1^ H_2_S treatment declined immediately and was the lowest on day 2 and then gradually increased until the end of storage (Figure 4B). The 15 μl L^−1^ H_2_S treatment group performed significantly lower than other treatments (*P* < 0.05). The 15 μl L^−1^ H_2_S treatment significantly alleviated the dramatic increase in PPO activity (Figure 4C) and maintained a lower level (*P* < 0.05). POD activity of all treatment groups increased gradually during storage. The 15 μl L^−1^ H_2_S treatment inhibited POD activity and was consistently lower than the control group (*P* < 0.05) (Figure 4D).

## Discussion

Browning of fresh-cut products is the result of mechanical damage induction during processing, which destroys the cell wall structure, activates phenolic metabolism, and leads to enzymatic browning (8, 27). In the present study, the increase of BI values of H_2_S-treated samples was significantly inhibited (Figure 1) and showed that H_2_S inhibited the browning. This result was consistent with fresh-cut lotus root slices treated by H_2_S (28). For the time period, we found that the control was corrupted on day 4 and that H_2_S treatment can significantly inhibit browning of fresh-cut CWCs. Fresh-cut CWCs of H_2_S treatment can still be well-presented until day 8 and then begin to lose commodity value.

ROS is an important factor causing enzymatic browning. Some experiments showed that the balance in the active oxygen metabolism system in the body is gradually destroyed after the products were cut. Although overproduction of ROS and oxidative damage are the universal events (11, 29), ${\text{O}}_{2}^{\cdot -}$ and H_2_O_2_ accelerate the senescence of fresh-cut fruits and vegetables during storage. Antioxidant enzymes are the most effective ROS scavengers; they can reduce the ROS levels in organisms and can delay browning (30). Accordingly, correlation analysis showed that there was a positive correlation between BI and ROS accumulation (*r* = 0.697–0.968) in the fresh-cut CWCs during 8-day storage (Table 1). Herein, antioxidant enzyme (CAT, SOD, APX, and GR) activities (Figure 3) in samples treated with H_2_S were significantly higher than their respective controls in maintaining dramatically low ${\text{O}}_{2}^{\cdot -}$ production rate and H_2_O_2_ content (Figure 2). Previous studies have shown that APX activity in protein extracts of *Arabidopsis thaliana* leaves treated with sulfide (NaHS) increased by 40%, and APX incubation with NaHS has been proven to be regulated through *S*-sulfhydrating (31). H_2_S as a treating agent is highly lipophilic, can freely pass through cell membrane, can react with sulfhydryl (–SH), and mediates important posttranslational modifications in *S*-sulfhydrating (persulfidation) (32). APX is the key enzyme responsible for H_2_O_2_ scavenging during oxidative stress in plants (33). In the current work, APX activity in H_2_S treatment remained higher all the time. However, correlation analysis showed that there was a negative correlation between BI and antioxidant enzyme activity. The result indicates that the inhibition by H_2_S on browning has nothing to do with the higher antioxidant enzyme activities in fresh-cut CWCs. Non-enzymatic antioxidants such as total phenolics also help to maintain a balanced ROS metabolism by quenching ROS (34) and can induce oxidative stress by inhibiting free radicals (35). Correlation analysis showed that there was a positive correlation between H_2_O_2_ content and total phenolics (*r* = 0.865, *P* < 0.01) in samples (Table 1). In the present study, H_2_S treatment is found to promote the increase of total phenolics and highlight the protective role of H_2_S in fresh-cut CWC storage. This showed that H_2_S treatment can maintain the metabolic balance of ROS in cells and reduce the accumulation of ROS burst by improving the activities of antioxidant enzymes and antioxidant defense system of the samples. In brief, the data proved that H_2_S treatment can delay the browning in fresh-cut CWCs by increasing the antioxidant enzyme activities and non-enzymatic antioxidant content that suppressed the accumulation of ROS (${\text{O}}_{2}^{\cdot -}$ and H_2_O_2_) and its associated oxidative damage to tissues.

**Table 1:** Pearson correlation coefficients of BI, H_2_O_2_ content, ${\text{O}}_{2}^{\cdot -}$ production rate, total phenol, and enzyme (CAT, SOD, APX, GR, PAL, PPO, and POD) activities of fresh-cut Chinese water chestnuts during storage.

**Trait**	***r*-value**										
	**BI**	**H**_**2**_**O**_**2**_	**$O_{2}^{\cdot -}$**	**CAT**	**SOD**	**APX**	**GR**	**Total phenol**	**PAL**	**PPO**	**POD**
BI	1	0.968^**^	0.697^*^	-0.859^**^	−0.692^*^	−0.833^**^	−0.741^**^	0.758^**^	0.789^**^	0.245	0.897^**^
H_2_O_2_		1	0.640^*^	-0.815^**^	−0.623^*^	−0.794^**^	−0.637^*^	0.865^**^	0.711^**^	0.06	0.949^**^
${\text{O}}_{2}^{\cdot -}$			1	-0.881^**^	−0.877^**^	−0.910^**^	−0.878^**^	0.294	0.837^**^	0.523	0.685^*^
CAT				1	0.878^**^	0.940^**^	0.929^**^	−0.542	−0.857^**^	−0.407	−0.880^**^
SOD					1	0.839^**^	0.925^**^	−0.356	−0.784^**^	−0.513	−0.670^*^
APX						1	0.907^**^	−0.485	−0.875^**^	−0.392	−0.843^**^
GR							1	−0.288	−0.843^**^	−0.597^*^	−0.699^*^
Total phenol								1	0.318	−0.373	0.817^**^
PAL									1	0.689^*^	0.739^**^
PPO										1	0.094
POD											1

*BI, browning index; CAT, catalase; SOD, superoxide dismutase; APX, ascorbate peroxidase; GR, glutathione reductase; PAL, phenylalanine ammonia lyase; PPO, polyphenol oxidase; POD, peroxidase*.

* *Significant at the P < 0.05 probability level*.

** *Significant at the P < 0.01 probability level*.

PAL is involved in the synthesis of free phenolics and catalyzes the oxidation of phenolics to brown pigments; cutting damage could accelerate the increase of PAL activity and surface browning of fresh-cut products (8, 36). Correlation analysis showed that there was a positive correlation between BI and PAL activity (*r* = 0.789, *P* < 0.01) in fresh-cut CWCs (Table 1). In the present study, PAL activity in H_2_S treatment increased slowly, indicating that H_2_S may inhibit PAL activity to inhibit enzymatic browning of sample surfaces. However, the higher level of total phenolic content in H_2_S fumigated samples and lower PAL activity seem like a contradiction and is similar to that of fresh-cut apples (37). How H_2_S regulated the activity of PAL and phenolic metabolism needs in-depth investigation. Browning enzymes are important food quality-related enzymes that are linked with changes in sensory and nutritional quality (38). POD participates in lignin formation in plants (39). Studies have found that sulfur compounds can competitively inhibit the activity of browning enzymes, thus inhibiting the occurrence of enzymatic browning (40). In another study, the increase of PPO and POD activities is inhibited by H_2_S treatment, alleviating whitening of fresh-cut carrots (8) and retarding the browning of fresh-cut lotus root slices during storage (28). The current study clearly elucidated that the H_2_S treatment inhibited the increase of PPO and POD activities (Figure 4), delaying the browning and maintaining the commodity value of fresh-cut CWCs. Correlation analysis showed that BI of fresh-cut CWCs was positively correlated with POD activity (*r* = 0.897, *P* < 0.01).

## Conclusions

In this study, the 15 μl L^−1^ H_2_S treatment could better inhibit browning of fresh-cut CWCs in complete storage. The higher total phenolic content and antioxidant enzyme activities of samples in the 15 μl L^−1^ H_2_S treatment made it possess stronger antioxidant capacity, thus inhibiting ROS accumulation and associated oxidative damage to fresh-cut CWCs. H_2_S effectively inhibited the increase in the activity of PAL, PPO, and POD. Correlation analysis showed that the browning inhibition by H_2_S was exerted by reducing ROS accumulation, increasing total phenolics, and inhibiting browning enzyme activity. It is speculated that H_2_S inhibits browning of fresh-cut CWCs by affecting antioxidant capacity and phenolic metabolism. Taken together, 15 μl L^−1^ of H_2_S had the potential to delay the senescence and maintain higher commodity value of fresh-cut CWCs.

## Data Availability Statement

The raw data supporting the conclusions of this article will be made available by the authors, without undue reservation.

## Author Contributions

YD: writing - original draft and formal analysis. CC: investigation. JW, ZC, WZ, HD, ZG, and CW: resources. JC: supervision. LZ: conceptualization writing - review and editing, and funding acquisition. All authors contributed to the article and approved the submitted version.

## Conflict of Interest

The authors declare that the research was conducted in the absence of any commercial or financial relationships that could be construed as a potential conflict of interest.
